# Perinatal effects of scorpion venoms: maternal and offspring development

**DOI:** 10.1186/s40409-017-0121-z

**Published:** 2017-06-14

**Authors:** Ana Leticia Coronado Dorce, Adriana do Nascimento Martins, Valquiria Abrão Coronado Dorce, Ana Leonor Abrahão Nencioni

**Affiliations:** 1 Laboratory of Pharmacology, Butantan Institute, Av. Dr. Vital Brasil, 1500, São Paulo, SP CEP 05503-900 Brazil; 2 Graduate Program in Sciences – Toxinology, Butantan Institute, Av. Dr. Vital Brasil 1500, São Paulo, SP CEP 05503-900 Brazil

**Keywords:** Scorpion venoms, Pregnancy, Lactation, Reproductive development, Perinatal development

## Abstract

Scorpion envenomation is a public health problem, especially in tropical and subtropical countries. Considering the high incidence of scorpionism in some areas, pregnant women and nursing mothers may be possible victims. Scorpion stings alter the release of neurotransmitters and some cytokines. These mediators act as organizers and programmers in the adequate formation of the nerves, and non-physiological concentrations of them during the brain organization originate disorders and diseases that can appear later in the life of the individual. Despite the importance of this subject, there are only a few studies showing the effects of scorpion venom on maternal reproductive development, in the morphology and physical and behavioral development of offspring. The present review article summarizes the major findings on this issue. Biochemical changes in the blood – such as hyperglycemia, increase on the level of sodium and on the creatinine concentration – are observed after scorpion sting in humans and experimental animals. Some studies in the literature demonstrate that the scorpion venom affects the maternal reproductive development in humans and in experimental animals, increasing the frequency and amplitude of uterine contraction and the number of resorptions. The venom can also lead to some alterations in the embryonic or fetal development increasing the total weight of fetuses and of some organs. Moreover, it affects the general activity and locomotion during childhood and adulthood, and the anxiety level in adult females and males. It also alters the number of hippocampal neurons and interferes in the level of some cytokines. Altogether, it is evident that the venom, when administered during the pregnancy or lactation, affects the development of the offspring. Studies are being conducted to determine the actual participation of the venom in the development of the offspring, and to what extent they are detrimental to animal development.

## Background

Scorpion envenomation is a public health issue in many places around the world, especially in tropical and subtropical countries, not only due to the high incidence of accidents, but also because the venom can induce morbidity in stung patients [1]. On account of the high incidence of scorpionism, pregnant women and nursing mothers may be possible victims of scorpion envenomation.

According to Isbister [2], despite the great number of scorpion accidents per year, most cases are minor, with localized pain and minimal systemic involvement. Pain is present in more than 95% of the envenomation cases whereas systemic manifestations are less frequent [3, 4].

Due to the variety of scorpions found worldwide, a range of clinical cases may occur [3]. Although some differences are observed, systemic envenomation is characterized by relatively similar neurotoxic excitation syndromes that result from the release of neurotransmitters and due to the action of neurotoxins on sodium channels [2–4]. Besides the release of neurotransmitters from the autonomic nervous system, scorpion venom may also provoke changes in the central nervous system, including epileptiform activity and neuronal damage in hippocampal neurons [5–10]. It is also responsible for tissue alterations, which lead to the release of inflammatory mediators such as cytokines [11–13].

Scorpion envenomation is classified based on signs and symptoms shown by the victim:

- mild – patients show local signs, such as edema, erythema, and sweating;
- moderate – nausea, abdominal pain, tachypnea, tachycardia or bradycardia, mild hypertension, agitation, hypersalivation, fever, priapism, and hyperglycemia are present;
- severe – characterized by cardiovascular complications (congestive heart failure, arrhythmia or severe hypertension); pulmonary complications (edema and respiratory distress syndrome); gastrointestinal complications (acute pancreatitis); metabolic complications (hyperglycemia, hypocalcemia, hyperkalemia or acid–base imbalance); and neurological symptoms (hypertensive encephalopathy, coma or convulsion) [4].

There are approximately 1,500 species of scorpions described worldwide and only about 30 of them belong to the Buthidae family, which is considered dangerous for humans and responsible for serious cases of envenomation and even death [7]. Some physiological alterations that occur during the envenomation caused by these scorpions can impair the proper development of the pregnancy and the fetus. For example, the maternal adequate oxygenation is of utmost importance to the fetus, as well the adequate level of glucose in the blood or the blood pressure [14].

Neurotransmitters and cytokines regulate essential processes of life. In addition, during the pre- and postnatal development, they act as organizers and programmers in the adequate formation of the nervous system [15]. Non-physiological concentrations of these mediators during the brain organization originate disorders and diseases that appear later in the life of the individual, which means they can act as endogenous teratogens [15].

During the development of the nervous system, neurotransmitters perform important roles [16]. The neuronal development is characterized by a combined action of trophic and degenerative processes leading to a complex organization of the mature central nervous system. This is a selection process in which certain neurons are stimulated to survive, while others are suppressed or even eliminated. Some evidence suggests that classical neurotransmitters are actively involved in these processes as promoters or suppressors of neurons [17]. Monoamines appear early in embryos, before the differentiation of neurons, and several studies have demonstrated that they are fundamental in the regulation of the brain development before assuming their role as neurotransmitters in the mature brain [18, 19]. Cytokines perform an important role in the development, proliferation, survival, differentiation, and growth, as well as in the neuronal regulation of neuronal synapses [20].

Few reports are documented in details in the literature regarding the effects of the scorpion venom in the perinatal phase, and the available data are somewhat controversial. Some authors state that a scorpion sting during pregnancy does not affect the mother or the fetus. In this sense, Langley [21], in an extensive review of studies published between 1966 and 2002, showed that no adverse consequence was found in human mothers – or their fetuses – when they were stung by a scorpion during the pregnancy. Similarly, Kaplanoglu and Helvaci [22] described the clinical findings of scorpion stings in 11 pregnant women. All patients developed mild envenomation and pregnancy complications were not observed. In a clinical case described by Bozkurt et al. [23], an early-stage pregnant woman stung by a yellow scorpion had excruciating pain without any other local or systemic complaints, and there were no consequences for the mother or the fetus. Ismail et al. [24] reported 52 cases of scorpionism, among those, two were pregnant women who presented no further consequences for them or their fetuses.

Although a retrospective study carried out in Tunisia from 1990 to 2004 with twenty pregnant women demonstrated neither maternal or fetal death, nor preterm fetal delivery, it reported two patients who manifested intense pelvic pain and one of them had vaginal bleeding [25]. Moreover, Ismail et al. [26] reported abortions in pregnant women whereas Zengrin et al. [27] observed seizure and eclampsia in a late-stage pregnant woman stung by a scorpion in Turkey. Kankonkar et al. [28] noted that a number of pregnant women died due to the red scorpion sting in India, but no details were provided. Finally, Leibenson et al. [29] reported a case of a pregnant woman that gave birth to a stillborn infant following a yellow scorpion sting.

Despite the importance of the subject, there are only a few studies showing the effects of the scorpion venom on the maternal reproductive development and on the morphology of the offspring, as well as on their physical and behavioral development. This review summarizes the major findings on this issue, mainly regarding the experimental studies. According to Brown et al. [14], the evidence regarding the maternal history and the treatment of scorpion envenomation during pregnancy is derived from animal data, and extrapolations to humans are limited due to differences in the study design and interspecies differences and responses to the envenomation.

## Effects of envenomation during pregnancy on prenatal development

### Hematologic changes in pregnancy

Throughout the course of the pregnancy until birth, the biological systems of females are deeply changed in order to ensure a good fetal development [30]. Any disorder may threaten both the mother and the child. Hyperglycemia and maternal hypertension, for example, generally contribute to embryonic and fetal malformations, as well as teratogenicity and fetal lethality [31].

Scorpion venom induces a body system response that leads to hyper- or hypotension, hypothermia, tachycardia, leukocytosis, hyperglycemia, myocarditis, pancreatitis, respiratory disorders and other complications, both in humans and in experimental animals [32]. Biochemical and hematological changes, as previously stated, such as hyperglycemia, hyperamylasemia, increase in serum activities of creatine kinase and aspartate aminotransferase and increase in hematocrits, red blood cells count, and in hemoglobin concentration have been observed after the scorpion stings in humans and experimental animals [4, 32–34].

Maternal blood alterations including hyperglycemia, increase in sodium and creatinine concentration were observed [35]. Ben Nasr et al. [31] showed that the *Buthus occitanus tunetanus* venom causes biological changes in the plasma of pregnant females, such as alterations in blood pressure and in the lipid peroxidation levels, and increase in the concentration of creatinine, β-estradiol and progesterone. Two pregnant women accidentally stung by scorpions presented hypertension associated with pelvic pain and vaginal bleeding. Neither maternal or fetal death nor preterm fetal delivery was observed [25]. Another reported case showed that a pregnant woman stung by a scorpion had discreet biochemical and physiological changes; however, during the period of observation, she was diagnosed with eclampsia [27]. The experimental injection of *B. o. tunetanus* venom in pregnant rats caused similar effects on maternal blood parameters, including increase in sodium level and reduction in glucose level, increase in the concentration of creatinine, urea and white blood cells [36]. These observations suggest that the envenomation may cause disturbances in the gestational process and in the development and well-being of the fetus [31].

### Alterations in the maternal reproductive system during pregnancy

Some studies suggest that the scorpion venom provokes alterations in the maternal reproductive system in humans and in experimental animals [13, 25, 31, 37–39].

It was shown that the *Leiurus quinquestriatus* venom increases the frequency and amplitude of uterine contractions mediated by the release of kinins [40]. Ismail et al. [26] showed that the *Androctonus amoreuxi* venom, injected repeatedly from the 5^th^ to the 8^th^ day of pregnancy, increases the number of fetal resorption and causes defects in ossification and weight loss in survivor pups. Moreover, *Buthus minax* venom causes skeletal malformations in goats and induces abortions in pregnant women [26]. *Buthus occitanus* venom contains a peptide that enhances bradykinin levels leading to muscle contraction in isolated uterus of rats, whereas T1 toxin from *Tityus serrulatus* venom causes the same effect [41, 42].

*L. quinquestriatus* venom produces a marked effect on the frequency and amplitude of uterine contractions of pregnant rats that seem to be more sensitive to the effects of the venom at the beginning of the pregnancy [43].

A study carried out with *T. serrulatus* venom demonstrated that a single injection on the 5^th^ or 10^th^ day of gestation in rats did not cause any significant change in the maternal reproductive system. However, two dams treated on the fifth day of gestation presented pre-implantation loss and other two had post-implantation loss without changes to maternal weight gain [37]. The same venom at a higher dose injected into females on the 10^th^ day of gestation increased the number of post-implantation losses [38]. Likewise, a single dose injection of *Tityus bahiensis* venom in pregnant rats on the 5^th^ or 10^th^ gestational day caused an increase in the number of pre-implantation losses of blastocysts [44].

Some studies demonstrated that scorpion venoms affect cytokines, which may be involved in the observed pregnancy losses [12, 33, 45]. Cytokines are important mediators in the gestational process, which lead to a successful pregnancy since they are involved in the implantation of the blastocyst, embryo formation and development, and especially in the development of the central nervous system [46]. The uterus promotes an immune adaptation during pregnancy to prevent maternal rejection [47]. The IL-10 cytokine seems to be the key to maintaining the gestational process due to this protection effect of the fetal-placental unit, inhibiting the secretion of inflammatory cytokines, such as IL-6 and INF-γ [48]. An interference of the venom with these mediators would be the cause of the alterations observed after the envenomation.

### Alterations in offspring morphology

In addition to affecting the appropriate development of pregnancy, scorpion venoms can also lead to some alterations in the embryonic or fetal development. *T. bahiensis* venom, when administered to rats on the 5^th^ or 10^th^ gestational day, caused changes in the offspring, such as increase in the fetus, placenta, lung, liver and heart weight, although the visceral macroscopic analysis demonstrated a normal appearance of the organs. The skeletal analysis of these pups showed no structural alteration [44]. Similarly, the venom of the *T. serrulatus* provoked an increase in the weight of the lungs, liver and placenta of fetuses of mothers who received a single dose of this venom during pregnancy [37]. Ben Nars et al. [49] showed a similar increase after the injection of the *B. o. tunetanus* venom. According to these authors, such effects are related to disabled placental function, maternal hypertension and metabolic disorders.

The augmented weight of pups is not traditionally considered fetal damage, but the increased weight of the liver, lungs and heart can be considered a consequence of the envenomation. Changes in the weight of pups, placenta and of some organs can be explained by alterations in the placental and embryonic pathway. Among the numerous metabolic processes of the placenta, many are considered essential for a good embryonic development. For the fetuses, the placenta is an all-in-one endocrine organ, that is, a combination of the gastrointestinal tract, kidneys, liver, lungs, spleen, and thymus. It carries on nutrients to the fetal circulation and is responsible for the metabolism of this system [50, 51]. An increase in the weight of the placenta could indicate changes to the metabolism of these nutrients that would be not necessarily a good or a bad thing for the pups [37]. The increase in fetal weight could be a consequence of an increase in the weight of the organs, or alternatively, an increase in the muscular mass or body fat of these pups [44].

The respiratory system appears to be particularly sensitive to the venom [52]. During envenomation, the lungs accumulate large quantities of scorpion venom [53]. The increase in pulmonary weight of the offspring could confirm the sensitivity of this organ to the venom. The mechanism by which the venom increases the weight of the lung in the offspring is not clear, but lung development can be changed in various ways, including by the release of glucocorticoids after the contact with the venom [54]. Cytokines appear to be clearly involved in the alterations found in the venom-treated offspring. Variations in the levels of IL-1 could be responsible for the increased weight of the placentas, since the IL-1 system is associated with the placental damage and increase in the brain [55].

## Effects of envenomation during pregnancy on postnatal development

### Physical and reflexological development

From birth to senescence, humans and animals develop a set of motor skills that ensure their survival and physical independence. Since pregnancy is a delicate phase in the formation of several biological systems, external or internal agents could generate fetal development disorders, damaging structures responsible for the physical, motor and behavioral development of the offspring.

Lactation is another important phase for the complete development of the offspring, during which the largest outbreak of brain growth and maturation of various organs and biological systems occur. The blood–brain barrier and the blood-gut barrier are still immature and depend on nutrients provided through breastfeeding for proper development [56, 57].

Some experimental studies have demonstrated that the injection of scorpion venom during pregnancy or lactation can induce alterations in the postnatal development. The injection of the *T. serrulatus* venom on the 10^th^ or 16^th^ gestational day causes an increase in the weight of the pups at birth, and alterations of some physical parameters including delay in ear opening and palmar grasp, and advance in the ear unfolding, and subtle changes in the motor reflexes [38]. *T. bahiensis* venom, experimentally administered on the same days, caused similar alterations, but with gender differences [58].

In another study, when *T. bahiensis* venom was injected into mothers during lactation on the 2^nd^, 10^th^ or 16^th^ days, subtle alterations were observed in the physical parameters of the pups [59]. Such variations included delay in ear opening, in the incisor tooth eruption and unfolding of the ear, as well as alterations in the postural reflexes, such as delay in the surface righting and negative geotaxis [59].

According to Martins et al. [59], the venom interferes, directly or indirectly, with the maturation of the systems or alters some processes, such as cellular differentiation or repair. The venom promotes the release of several inflammatory mediators, among them, IL-6 [11]. It has already been demonstrated that prenatal administration of IL-6 increases the birth weight, since it reaches the fetus through the placenta [60, 61].

### Behavioral development

The evolution of the locomotion, from pregnancy until adolescence, does not involve a single system, but it depends on a variety of systems, such as the sensory, motor, and postural systems and neurotransmitters [62]. During the long period of neural maturation there are critical periods that are subject to internal and external influences, inducing various behavioral effects [63]. Scorpion venom can be included among the external factors that lead to behavioral changes in the offspring.

In this sense, it was demonstrated that the administration in rats of *T. bahiensis* venom on 16^th^ day of pregnancy increases the general activity and locomotion in the male offspring during adulthood, and locomotion in pups [58]. On the other hand, the injection of the same venom on the 10^th^ day of pregnancy decreases the general and locomotor activities of males during adulthood [39]. During lactation, depending on the time of application of the venom, there is a subtle increase or decrease of this behavior, in both males and females, in childhood and adulthood [59].

According to Vinay et al. [64], in rats, the peak of the motor development takes place around the second week of life. The direct or indirect action of the venom would be responsible for injuries in the formation of motor pathways or, alternatively, for an unbalance in the release of important neurotransmitters, such as dopamine, which is critical for the control of the motor activity [63, 65]. Females are more susceptible to prenatal stress [66]. Maternal proinflammatory cytokines are associated to fetal cerebral injuries [67–69]. Immune reactions mediated by cytokines during the embryonic period or during childhood can increase the risk of cognitive and/or psycho-behavioral mismatches [70]. The administration of *T. bahiensis* venom during pregnancy decreases the level of anxiety in males during adulthood, while in females it is increased [39]. During lactation, the anxiety level in females is decreased, but alterations in emotionality were observed in both genders [59].

According to Mathews [71], anxiety and memory are intrinsically related, and anxiety is necessary for the memory formation. In the case of the venom, it is possible that emotionality and cognition are affected by different routes within the nervous system, since there is no evidence of any damage to the formation and consolidation of the memory.

### Structural alterations to the hippocampus

The hippocampus has an important participation in the emotional cognitive control and in motivational processes [72]. Due to its evident importance, histological studies were conducted in order to evaluate whether this structure is affected by the venom, and whether there is any correlation with the physical, reflexological and behavioral alterations caused by the venom [39, 59].

*T. bahiensis* venom, when administered to pregnant rats, causes neuronal loss in the CA1, CA3 and CA4 hippocampal areas of the offspring [39]. On the other hand, when applied during lactation, an increase in the number of hippocampal neurons in pups and in adults was observed [59]. Several studies indicate that prenatal stress causes a significant reduction in the cellular proliferation; however, depending on the intensity of the stress, this neurogenesis can be suppressed or reinforced in specific cerebral areas [73, 74]. During the perinatal phase, an exponential growth of the brain takes place. More than 85% of the hippocampal neurons are formed during this period and, at the end of the first week, a physiological death of approximately 70% of the neurons occurs [75]. The peripheral activation of T cells is associated with an increase in the cellular proliferation and hippocampal neurogenesis [76]. The offspring of mothers treated with the venom during pregnancy or lactation had an increase on the level of cytokines [13, 59].

### Cytokines

It was demonstrated that *T. bahiensis* venom injection during pregnancy decreases the level of INF-γ, IL-10 and TNF-α in the fetuses 24 h after the application, and it increases the level of IL-1α six hours after the application [13]. When the injection occurs during the lactation period, there is an increase in the cerebral level of INF-γ in pups 24 h after the maternal envenomation [59].

During pregnancy, cytokines take part on the blastocyst implantation, formation and development of the embryo [46]. During the perinatal period, cytokines participate in the development of the central nervous system, and are extremely important for the neurogenesis, apoptosis and control of neuronal processes [77]. According to Lin et al. [48], IL-10 is responsible for maintaining the pregnancy and for protecting the placenta-fetal unit. The implantation losses observed after envenomation could be explained by a reduction on the level of IL-10 [38, 44].

The activation of the immune system, particularly IFN-γ, is associated with neurogenesis [78–80]. High levels of proinflammatory cytokines generated by the mother or the fetal immune system are associated with abnormal fetal brain development and neurological disorders that may be responsible for unexplained behaviors occurring in adulthood [81].

From the gestation until approximately the second week of life, the development presents several windows in the maturation processes that can suffer damages by internal and external factors [82]. The changes induced by the venom on cytokine levels during gestation or lactation could be related to the alterations in the physical, cognitive and behavioral development of the offspring [39, 58, 59].

## Conclusions

Altogether, it is evident that scorpion venom, when administered during pregnancy or lactation in animal models, affects the development of the offspring. However, further studies in this area are necessary in order to determine the actual participation of the venom in the development of the offspring, and to what extent it is detrimental to the animal development. Moreover, the mechanisms of action and the systems affected by scorpion venoms during the perinatal phase should be the subject of further studies, as well as the pharmacokinetics during pregnancy.

## References

[1] Goyffon M (2002). Le scorpionisme. Rev Fr Lab.

[2] Isbister GK (2014). Scorpion envenomation. N Engl J Med.

[3] Chippaux JP (2012). Emerging options for the management of scorpion stings. Drug Des Devel Ther.

[4] Santos MSV, Silva CGL, Silva Neto B, Grangeiro Junior CRP, Lopes VHG, Teixeira Junior AG (2016). Clinical and epidemiological aspects of scorpionism in the world: a systematic review. Wilderness Environ Med.

[5] Teixeira AL, Fontoura BF, Freire-Maia L, Machado CRS, Camargos ERS, Teixeira MM. Evidence for a direct action of *Tityus serrulatus* scorpion venom on the cardiac muscle. Toxicon. 2001;39(5):703–9. 10.1016/s0041-0101(00)00200-2 11072050

[6] de Sousa Alves R, do Nascimento NRF, Barbosa PSF, Kerntopf MR, Lessa LMA, Sousa CM (2005). Renal effects and vascular reactivity induced by *Tityus serrulatus* venom. Toxicon.

[7] Chippaux JP, Goyffon M (2008). Epidemiology of scorpionism: a global appraisal. Acta Trop.

[8] Lourenço GA, Lebrun I, Coronado VA (2002). Neurotoxic effects of fractions isolated from *Tityus bahiensis* scorpion venom (Perty, 1834). Toxicon.

[9] Nencioni ALA, Lourenço GA, Lebrun I, Florio JC, Dorce VAC (2009). Central effects of *Tityus serrulatus* and *Tityus bahiensis* scorpion venoms after intraperitoneal injection in rats. Neurosci Lett.

[10] Ossanai LTT, Lourenço GA, Nencioni ALA, Lebrun I, Yamanouye N, Dorce VAC (2012). Effects of a toxin isolated from *Tityus bahiensis* scorpion venom on the hippocampus of rats. Life Sci.

[11] Fukuhara YDM, Reis ML, Dellalibera-Joviliano R, Cunha FQC, Donadi EA (2003). Increased plasma levels of IL-1β, IL-6, IL-8, IL-10 and TNF-α in patients moderately or severely envenomed by *Tityus serrulatus* scorpion sting. Toxicon.

[12] Petricevich VL (2010). Scorpion venom and the inflammatory response. Mediators Inflamm.

[13] Dorce ALC, Frare EO, Paulo MEFV, Dorce VAC, Nencioni ALA (2015). *Tityus bahiensis* scorpion venom injected to dams during pregnancy affects some cytokines of fetuses. Toxicon.

[14] Brown SA, Seifert SA, Rayburn WF (2013). Management of envenomations during pregnancy. Clin Toxicol (Phila).

[15] Dörner G, Plagemann A (2012). Perinatal brain programming and functional teratology. Perinatal programming – the state of the art.

[16] Rice D, Barone S (2000). Critical periods of vulnerability for the developing nervous system: evidence from humans and animal models. Environ Health Perspect.

[17] Meier E, Hertz L, Schousboe A (1991). Neurotransmitters as developmental signals. Neurochem Int.

[18] Levitt P, Harvey JA, Friedman E, Simansky K, Murphy EH (1997). New evidence for neurotransmitter influences on brain development. Trends Neurosci.

[19] Whitaker-Azmitia PM (2001). Serotonin and brain development: role in human developmental diseases. Brain Res Bull.

[20] Gilmore JH, Jarskog LF, Vadlamudi S (2003). Maternal infection regulates BDNF and NGF expression in fetal and neonatal brain and maternal-fetal unit of the rat. J Neuroimmunol.

[21] Langley RL (2004). A review of venomous animal bites and stings in pregnant patients. Wilderness Environ Med.

[22] Kaplanoglu M, Helvaci MR (2015). Scorpion stings in pregnant women: an analysis of 11 cases and review of literature. Clin Exp Obstet Gynecol.

[23] Bozkurt S, Okumus M, Utku B, Arikan DC, Lok U, Duygu FB (2015). Scorpion sting in a pregnant woman: a case report. Arch Clin Exp Surg.

[24] Ismail M, Abd-Elsalam A (1994). al-Ahaidib MS. *Androctonus crassicauda* (Olivier), a dangerous and unduly neglected scorpion--I. Pharmacological and clinical studies. Toxicon.

[25] Ben Nasr H, Hammami TS, Sahnoun Z, Rebai T, Bouaziz M, Kassis M (2007). Scorpion envenomation symptoms in pregnant women. J Venom Anim Toxins incl Trop Dis.

[26] Ismail M, Ellison AC, Tilmisany AK (1983). Teratogenicity in the rat of the venom from the scorpion *Androctonus amoreuxi* (Aud. & Sav.). Toxicon.

[27] Zengin S, Al B, Oktay MM, Kilic H. Scorpion sting: eclampsia. BMJ Case Rep. 2012;7(2012):pii bcr1220115401. doi: 10.1136/bcr.12.2011.5401. 10.1136/bcr.12.2011.5401 PMC3448761 22962387

[28] Kankonkar RC, Kulkurni DG, Hulikavi CB (1998). Preparation of a potent anti-scorpion-venom-serum against the venom of red scorpion (*Buthus tamulus*). J Postgrad Med.

[29] Leibenson L, Leibenson M, Silberstein T (2010). Antepartum fetal death following a yellow scorpion sting. Arch Gynecol Obstet.

[30] Girling JC (2004). Physiology of pregnancy. Anaesth Intens Care Med.

[31] Ben Nasr H, Serria H, Chaker S, Riadh B, Zouheir S, Kamel J (2009). Some biological effects of scorpion envenomation in late pregnant rats. Exp Toxicol Pathol.

[32] D’Suze G, Moncada S, González C, Sevcick C, Aguilar V, Alagón A (2003). Relationship between plasmatic levels of various cytokines, tumor necrosis factor, enzymes, glucose and venom concentration following *Tityus* scorpion sting. Toxicon.

[33] D’Suze G, Salazar V, Díaz P, Sevcik C, Azpurua H, Bracho N (2004). Histopathological changes and inflammatory response induced by *Tityus discrepans* scorpion venom in rams. Toxicon.

[34] Cusinato DA, Souza AM, Vasconcelos F, Guimarães LF, Leite FP, Gregório ZM (2010). Assessment of biochemical and hematological parameters in rats injected with *Tityus serrulatus* scorpion venom. Toxicon.

[35] Nasr HB, Bolon B, Hammami ST, Sahnoun Z, Jamoussi K, Lahyani A (2009). Clinical pathology alterations in pregnant and non-pregnant rats following scorpion envenomation. Basic Clin Pharmacol Toxicol.

[36] Ben Nasr H, Hammami S, Mion G, Sahnoun Z, Chouaiekh F, Rebai T (2007). Effects of *Buthus occitanus tunetanus* envenomation on an experimental murine model of gestation. C R Biol.

[37] Cruttenden K, Nencioni ALA, Bernardi MM, Dorce VAC (2008). Reproductive toxic effects of *Tityus serrulatus* scorpion venom in rats. Reprod Toxicol.

[38] Barão AAS, Nencioni ALA, Coronado VA (2008). Embryotoxic effects of maternal exposure to *Tityus serrulatus* scorpion venom. J Venom Anim Toxins incl Trop Dis.

[39] Dorce ALC, Dorce VAC, Nencioni ALA (2010). Effects of in utero exposure to *Tityus bahiensis* scorpion venom in adult rats. Neurotoxicol Teratol.

[40] Osman OH, Ismail M, el-Asmar MF, Ibrahim SA (1972). Effect on the rat uterus of the venom from the scorpion *Leiurus quinquestriatus*. Toxicon.

[41] Meki AR, Nassar AY, Rochat H (1995). A bradykinin-potentiating peptide (peptide K12) isolated from the venom of Egyptian scorpion *Buthus occitanus*. Peptides.

[42] Mendonça M, Luz MM, Freire-Maia L, Cunha-Melo JR (1995). Effect of scorpion toxin from *Tityus serrulatus* on the contraction of the isolated rat uterus. Toxicon.

[43] Marei ZA, Ibrahim SA (1979). Stimulation of rat uterus by venom from the scorpion *L. quinquestriatus*. Toxicon.

[44] Dorce ALC, Dorce VAC, Nencioni ALA (2014). Mild reproductive effects of the *Tityus bahiensis* scorpion venom in rats. J Venom Anim Toxins incl Trop Dis.

[45] Magalhães MM, Pereira MES, Amaral CFS, Rezende NA, Campolina D, Bucaretchi F (1999). Serum levels of cytokines in patients envenomed by *Tityus serrulatus* scorpion sting. Toxicon.

[46] Mousa A, Bakhiet M (2013). Role of cytokine signaling during nervous system development. Int J Mol Sci.

[47] Nan CL, Lei ZL, Zhao ZJ, Shi LH, Ouyang YC, Song XF (2007). Increased Th1/Th2 (INF-γ/IL-4) cytokine mRNA ratio of rat embryos in the pregnant mouse uterus. J Reprod Dev.

[48] Lin H, Mosmann TR, Guilber L, Tuntipopipat S, Wegmann T (1993). Synthesis of T helper 2-type cytokines at the maternal-fetal interface. J Immunol.

[49] Ben Nasr H, Badraoui R, Serria H, Zeghal K (2012). Embryotoxicity following repetitive maternal exposure to scorpion venom. J Venom Anim Toxins incl Trop Dis.

[50] Gude NM, Roberts CT, Kalionis B, King RG (2004). Growth and function of the normal human placenta. Thromb Res.

[51] Myllynen P, Pasanen M, Pelkonen O (2005). Human placenta: a human organ for developmental toxicology research and biomonitoring. Placenta.

[52] De-Matos IM, Talvani A, Rocha OO, Freire-Maia L, Teixeira MM (2001). Evidence for a role of mast cells in the lung edema induced by *Tityus serrulatus* venom in rats. Toxicon.

[53] Nunan EA, Moraes MF, Cardoso VN, Moraes-Santos T (2003). Effect of age on body distribution of Tityustoxin from *Tityus serrulatus* scorpion venom in rats. Life Sci.

[54] Newman LM, Johnson EM, Johnson EM, Kochhar DM (1983). Abnormal lung function induced by prenatal insult. Teratogenesis and Reproductive Toxicology.

[55] Girard S, Tremblay L, Lepage M, Sébire G (2010). IL-1 receptor antagonist protects against placental and neurodevelopmental defects induced by maternal inflammation. J Immunol.

[56] Murtazina AR, Nikishina YO, Bondarenko NS, Sapronova AJ, Ugrumov MV (2016). Signal molecules during the organism development: central and peripheral sources of noradrenaline in rat on ontogenesis. Dokl Biochem Biophys.

[57] Moussaoui N, Braniste V, Ait-Belgnaoui A, Gabanou M, Sekkal S, Olier M (2014). Changes in intestinal glucocorticoid sensitivity in early life shape the risk of epithelial barrier defect in maternal-deprived rats. PLoS One.

[58] Dorce AL, Bellot RG, Dorce VA, Nencioni AL (2009). Effects of prenatal exposure to *Tityus bahiensis* scorpion venom on rat offspring development. Reprod Toxicol.

[59] Martins AN, Nencioni ALA, Dorce ALC, Paulo MEFV, Frare EO, Dorce VAC (2016). Effect of maternal exposure to *Tityus bahiensis* scorpion venom during lactation on the offspring of rats. Reprod Toxicol.

[60] Samuelsson AM, Öhrn I, Dahlgren J, Eriksson E, Angelin B, Folkow B (2004). Prenatal exposure to interleukin-6 results in hypertension and increased hypothalamic-pituitary-adrenal axis activity in adult rats. Endocrinology.

[61] Dahlgren J, Samuelsson AM, Jansson T, Holmäng A (2006). Interleukin-6 in the maternal circulation reaches the rat fetus in mid-gestation. Pediatr Res.

[62] Swann HE, Kempe RB, Van Orden AM, Brumley MR (2016). Serotonergic activation of locomotor behavior and posture in one-day old rats. Behav Brain Res.

[63] Money KM, Stanwood GD (2013). Developmental origins of brain disorders: roles for dopamine. Front Cell Neurosci.

[64] Vinay L, Brocard F, Pflieger JF, Simeoni-Alias J, Clarac F (2000). Perinatal development of lumbar motoneurons and their inputs in the rat. Brain Res Bull.

[65] Aksu I, Baykara B, Ozbal S, Cetin F, Sisman AR, Dayi A (2012). Maternal treadmill exercise during pregnancy decreases anxiety and increases prefrontal cortex VEGF and BDNF levels of rat pups in early and late periods of life. Neurosci Lett.

[66] Zagron G, Weinstock M (2006). Maternal adrenal hormone secretion mediates behavioural alterations induced by prenatal stress in male and female rats. Behav Brain Res.

[67] Dinarello CA (2000). Proinflammatory cytokines. Chest.

[68] Duggan PJ, Maalouf EF, Watts TL, Sullivan MHF, Counsell SJ, Allsop J (2001). Intrauterine T-cell activation and increased proinflammatory cytokine concentrations in preterm infants with cerebral lesions. Lancet.

[69] Maleb S, Dammann O (2009). Fetal inflammatory response and brain injury in the preterm newborn. J Child Neurol.

[70] Tohmi M, Tsuda N, Watanabe Y, Kakita A, Nawa H (2004). Perinatal inflammatory cytokine challenge results in distinct neurobehavioral alterations in rats: implication in psychiatric disorders of developmental origin. Neurosci Res.

[71] Mathews A (1990). Why worry? The cognitive function of anxiety. Behav Res Ther.

[72] White AM, Matthews DB, Best PJ (2000). Ethanol, memory, and hippocampal function: a review of recent findings. Hippocampus.

[73] Lemaire V, Koehl M, Le Moal M, Abrous DN (2000). Prenatal stress produces learning deficits associated with an inhibition of neurogenesis in the hippocampus. Proc Natl Acad Sci U S A.

[74] Fujioka A, Fujioka T, Ishida Y, Maekawa T, Nakamura S (2006). Differential effects of prenatal stress on the morphological maturation of hippocampal neurons. Neuroscience.

[75] Bandeira F, Lent R, Herculano-Houzel S (2009). Changing numbers of neuronal and non-neuronal cells underlie postnatal brain growth in the rat. Proc Natl Acad Sci U S A.

[76] Wolf SA, Steiner B, Akpinarli A, Kammertoens T, Nassenstein C, Braun A (2009). CD4-positive T lymphocytes provide a neuroimmunological link in the control of adult hippocampal neurogenesis. J Immunol.

[77] Green HF, Nolan YM (2014). Inflammation and the developing brain: consequences for hippocampal neurogenesis and behavior. Neurosci Biobehav Rev.

[78] Yirmiya R, Goshen I (2011). Immune modulation of learning, memory, neural plasticity and neurogenesis. Brain Behav Immun.

[79] Baron R, Nemirovsky A, Harpaz I, Cohen H, Owens T, Monsonego A (2008). IFN-gamma enhances neurogenesis in wild-type mice and in a mouse model of Alzheimer’s disease. FASEB J.

[80] Butovsky O, Ziv Y, Schwartz A, Landa G, Talpalar AE, Pluchino S (2006). Microglia activated by IL-4 or IFN-gamma differentially induce neurogenesis and oligodendrogenesis from adult stem/progenitor cells. Mol Cell Neurosci.

[81] Meyer U, Feldon J, Fatemi SH (2009). *In-vivo* rodent models for the experimental investigation of prenatal immune activation effects in neurodevelopmental brain disorders. Neurosci Biobehav Rev.

[82] Heyer DB, Meredith RM (2017). Environmental toxicology: sensitive periods of development and neurodevelopmental disorders. Neurotoxicology.

